# Effects of BARLEYmax and high-β-glucan barley line on short-chain fatty acids production and microbiota from the cecum to the distal colon in rats

**DOI:** 10.1371/journal.pone.0218118

**Published:** 2019-06-11

**Authors:** Seiichiro Aoe, Chiemi Yamanaka, Miki Fuwa, Taiga Tamiya, Yasunori Nakayama, Takanori Miyoshi, Eiichi Kitazono

**Affiliations:** 1 Studies in Human Life Sciences, Graduate School of Studies in Human Culture, Otsuma Women’s University, Chiyoda-ku, Tokyo, Japan; 2 The Institute of Human Culture Studies, Otsuma Women’s University Chiyoda-ku, Tokyo, Japan; 3 TEIJIN Limited, Chiyoda-ku, Tokyo, Japan; National Institute for Agronomic Research, FRANCE

## Abstract

We investigated whether supplementation with the barley line BARLEYmax (*Tantangara*; BM), which contains three fermentable fibers (fructan, β-glucan, and resistant starch), modifies the microbiota in cecal and distal colonic digesta in addition to short-chain fatty acids (SCFAs) production more favorably than supplementation with a high-β-glucan barley line (BG012; BG). Male Sprague–Dawley rats were randomly divided into 3 groups that were fed an AIN-93G-based diet that contained 5% fiber provided by cellulose (control), BM or BG. Four weeks after starting the respective diets, the animals were sacrificed and digesta from the cecum, proximal colon and distal colon were collected and the SCFA concentrations were quantified. Microbiota in the cecal and distal colonic digesta were analyzed by 16S rRNA sequencing. The concentrations of acetate and *n*-butyrate in cecal digesta were significantly higher in the BM and BG groups than in the control group, whereas the concentration of total SCFAs in cecal digesta was significantly higher only in the BM group than in the control group. The concentrations of acetate and total SCFAs in the distal colonic digesta were significantly higher only in the BM group than in the control group. The abundance of *Bacteroidetes* in cecal digesta was significantly higher in the BM group than in the control group. In contrast, the abundance of Firmicutes in cecal digesta was significantly lower in the BM and BG groups than in the control group. These results indicated that BM increased the concentration of total SCFAs in the distal colonic digesta. These changes might have been caused by fructan and resistant starch in addition to β-glucan. In conclusion, fermentable fibers in BM reached the distal colon and modified the microbiota, leading to an increase in the concentration of total SCFAs in the distal colonic digesta, more effectively compared with the high-β-glucan barley line (BG).

## Introduction

Epidemiological studies have reported that the consumption of whole grain cereals may increase the bacterial fermentation of dietary fiber to short-chain fatty acids (SCFAs) which have anti-carcinogenic properties, and thereby reduce the risk of colonic disorders [1–3]. A recent systematic review concluded that high-fiber, whole grain cereals can improve bowel function [4]. Microbiota-accessible carbohydrates (MACs) found in dietary fiber were suggested to play a key role in shaping the microbial ecosystem in the gut [5]. That study showed that a diet low in such carbohydrates resulted in a progressive loss of microbiota diversity in gnotobiote mice inoculated by human microbiota. It has been demonstrated that the lack of dietary MACs reduces mucosal thickness of the distal colon, making it easy for intestinal microbes to invade the epithelium and increasing the colonic inflammatory state in mice [6]. Dysbiosis, which is defined as a disturbance of the normal functions of gut microbiota, can arise from several alterations to the microbiota including reduced bacterial diversity, an expansion of pathological bacteria, a change in the microbial composition, and a change in microbial functional capacity [7]. However, a recent systematic review and meta-analysis concluded that fiber intervention has no significant effect on the α-diversity of gut microbiota [8]. This discrepancy might have been caused by the different sources of dietary fiber used by the studies included in the review.

Soluble dietary fiber is expected to reduce the risk of dysbiosis via bacterial fermentation. The typical soluble fiber in whole grain foods is β-glucan. Barley β-glucans were shown to be easily fermented by the bacterial genera *Bacteroides* and *Prevotella* in an *in vitro* study [9]. We previously reported that the intake of high-β-glucan barley increased the abundance of *Bacteroides* as compared to β-glucan-free barley, whereas it decreased the abundance of *Clostridium* clusters in diet-induced obese mice [10]. Fructans, which include inulin in chicory and burdock, are also a class of fermentable dietary fibers that have been well studied and are clearly effective in stimulating the growth of health-promoting species belonging to the genera *Bifidobacterium spp*. and *Lactobacillus spp*. in humans [11]. On the other hand, insoluble dietary fibers such as cellulose have poor and slow fermentation characteristics [12]. However, resistant starch, which is an insoluble fiber, is well fermented in the colon [13]. Therefore, the fermentation of dietary fibers in the large intestine may be influenced by the chemical characteristics of the fiber, including sugar and linkage composition, and molecular size rather than whether it is soluble or insoluble [14].

A recent study reported that study participants fed BARLEYmax (BM) containing β-glucan, fructan, and resistant starch presented higher distal colonic output and defecation frequency than those who were fed the placebo cereal bar in a human study [15]. Intake of BM resulted in a significant increase in the production of SCFAs, an increase in the abundance of *Bacteroides*, and a decrease in the abundance of *Clostridium subcluster XIVa* [15]. The combination of multiple types of dietary fiber that have different fermentation rates has recently been a topic of interest. It was reported that a combination of several indigestible carbohydrates may affect both the profile of SCFAs produced by fermentation and the site of SCFA release in the rat hindgut [16]. It has been reported that β-glucans in barley have preventive effects on colonic disorders, and these are partially mediated by SCFA production [17]. Therefore, high-β-glucan barley such as BG is considered as more favorable for healthy colonic conditions compared with ordinary barley. On the other hand, BM contains three fermentable fibers, i.e., fructan, β-glucan, and resistant starch, that have different fermentation speeds. Therefore, two types of barley lines, BM and BG, were selected for the present study.

The purpose of the study was to investigate whether supplementation with BM, which contains several types of fermentable dietary fibers including fructan, β-glucan, and resistant starch, modifies the distal colonic microbiota more favorably than supplementation with BG, which contains a higher amount of β-glucan but lower amounts of fructan and resistant starch than BM. The fermentation speeds of fructans, β-glucans, and resistant starches are quite different. Fructans have the fastest fermentation speed and resistant starches have the slowest speed, while β-glucans have an intermediate speed. Stepwise fermentation of BM may cause SCFA production from the cecum to the distal colon. On the other hand, the major fermentable fiber in BG is β-glucan. We speculated that carbohydrate complexes of fructan, β-glucan, and resistant starch in BM are more widely fermented from the cecum to distal colon compared with β-glucan-dominant barley. To elucidate this hypothesis, we compared the fermentation characteristics between BM and BG from the cecum to the distal colon of Sprague-Dawley rats.

## Materials and methods

### Sample preparation and chemical analysis

BARLEYmax (Tantangara; BM) and BG012 (BG) were obtained from Teijin Limited (Tokyo, Japan). BM is a barley line developed by CSIRO (Commonwealth Scientific and Industrial Research Organisation) in Australia. BG is a high-β-glucan barley line that is a hulless, six-rowed, waxy endosperm barley [18]. This barley was selected from the cross of two varieties [18]. The total dietary fiber contents in BM and BG were analyzed by the method of Prosky et al. (AOAC 991.43) [19]. The β-glucan contents in BM and BG were measured by the method of McCleary et al. (AOAC 995.16) [20]. The fructan contents in BM and BG were analyzed using the method of AOAC 999.03 [21]. The resistant starch contents in BM and BG were analyzed using the method of AOAC 2002.02 [22]. The amounts of dietary fiber components in BM and BG are shown in Table 1. The nutrient components were analyzed by the Japan Food Research Laboratories (Tokyo, Japan). The amounts of total dietary fibers and β-glucan in BM were lower than those in BG, whereas the amounts of fructan and resistant starch were higher in BM than in BG. BM had a wider variation of dietary fiber components compared with BG. Other dietary fiber components in BG are considered to be arabinoxylan and cellulose which constitute the plant cell wall (the amounts of arabinoxylan and cellulose were not determined).

**Table 1:** Amounts of dietary fiber components in BARLEYmax and high-β-glucan barley (BG012).

Dietary fiber components	BM	BG
Total dietary fiber (g/100g)	16.4	18.8
β-glucan (g/100g)	6.3	9.0
Fructan (g/100g)	9.0	2.2
Resistant starch (g/100g)	3.0	0.5

BM: BARLEYmax; BG: BG012

### Animals and study design

Four-week-old Sprague–Dawley rats were purchased from Charles River Laboratories Japan, Inc. (Yokohama, Japan). The rats were individually housed in stainless steel wire cages. The room temperature and relative humidity were maintained at 23–26 °C and 40–60%, respectively, on a 12-h light/12-h dark cycle (the light was switched on at 08:00 h). This study was approved by the Otsuma Women’s University Animal Research Committee (Tokyo, Japan) and was performed in accordance with the Regulation on Animal Experimentation at Otsuma Women’s University. After acclimatization for 7 d, the rats were randomized to 3 groups (*n* = 8 per group) stratified by body weight and shifted to an AIN-93G-based experimental diet [23]. The control diet was supplemented with 5% cellulose. The BM and BG diets were supplemented with BM and BG powder each corresponding to 5% of total dietary fiber, respectively. The protein and fat contents in the BM and BG diets were adjusted with casein and soybean oil, respectively, to be the same as those in the control diet. The compositions of the experimental diets are shown in Table 2. Rats were fed the experimental diets *ad libitum* for 4 weeks. Food intake and body weight were monitored every day throughout the study period. The food efficiency ratio was calculated as the percentage of the ratio of body weight gain (g) per food intake (g).

Foodefficiencyratio=totalbodyweightgainatendofstudy(g)/totalfoodintakeduringthestudy(g)x100%

**Table 2:** Composition of the test diets (g/kg diet).

	Control diet	BM diet	BG diet
Corn starch	397.5	215.7	232.6
Dextrinized starch	132	132	132
Casein	200	147.9	159.6
Sucrose	100	100	100
Soybean oil	70.0	49.0	59.4
Cellulose	50	-	-
BM	-	304.9	-
BG	-	-	266.0
AIN-93G mineral mixture	35	35	35
AIN-93 vitamin mixture	10	10	10
ʟ-cystine	3	3	3
Choline bitartrate	2.5	2.5	2.5
*t*-butylhydroquinone	0.014	0.014	0.014

BM, BARLEYmax; BG, high-β-glucan barley (BG012).

Fresh distal colonic digesta (feces) were collected between 9:30 and 10:30 in the morning on day 26–27 of the study. At the end of the study, rats were euthanized by isoflurane/CO_2_ euthanasia without fasting. The cecum, proximal colon, and distal colon along with their digesta were dissected. The digesta in the cecum, digesta in the proximal colon, and digesta in the distal colon were collected and kept at −20 °C until analysis of SCFAs.

### Short-chain fatty acids analysis in the cecal, proximal colonic, and distal colonic digesta

The concentration of SCFAs in the cecal, proximal colonic, and distal colonic digesta was determined by gas chromatography-mass spectrometry as described previously [24]. The cecal, proximal colonic, and distal colonic digesta (10 mg per sample) were disrupted using 5-mm stainless beads (AS ONE Corp., Osaka, Japan) and homogenized in extraction solution containing 100 μl of internal standard (100 μM crotonic acid), 50 μl of HCl, and 200 μl of ether. After vigorous shaking using a TissueLyser II (Qiagen, Hilden, Germany) at 2,000 rpm for 15 min, the homogenates were centrifuged at 1,000 x g at 25 °C for 10 min and then the top ether layer was collected and transferred into new glass vials. Aliquots (80 μl) of the ether extracts were mixed with 16 μl of *N*-tert-butyldimethylsilyl-*N*-methyltrifluoroacetamide. The vials were sealed tightly by screwing the cap, heated at 80 °C for 20 min in an incubator, and then left at room temperature for 48 h for derivatization. The derivatized samples were run through a 5977A GC/MS instrument (Agilent, Tokyo, Japan) fitted with a DB-5MS column (30 m × 0.53 mm) (Agilent). The initial oven temperature was held at 60 °C for 3 min, ramped to 120 °C at a rate of 5 °C/min and then to 300 °C at a rate of 20 °C/min, and finally held at 300 °C for 2 min. Helium was used as a carrier gas at a constant flow rate of 1.2 mL/min. The temperatures of the front inlet, transfer line, and electron impact ion source were set at 250, 260, and 230 °C, respectively. The mass spectral data were collected in selective ion monitoring mode. The concentrations of the SCFAs were quantified by comparing their peak areas with the internal standards and expressed as μmol/g of the digesta of the cecum, proximal colon, and distal colon.

### Quantification of cecal and distal colonic microbiota by 16S rRNA sequencing

Total DNA in cecal and distal colonic digesta was extracted using a DNA stool kit (Qiagen, Hilden, Germany).

The V3–V4 region of the bacterial 16S rRNA was amplified from the DNA samples, and a library was constructed with two-step tailed polymerase chain reaction (PCR). Fragments of 16S rRNA were amplified from the DNA samples by PCR using 1st-341f_MIX (5′- Seq A—TCT TCC GAT CT—NNNNN—CCT ACG GGN GGC WGC AG -3′) [25] and 1st-805r_MIX (5′- Seq B—CTC TTC CGA TCT—NNNNN–GAC TAC HVG GGT ATC TAA TCC -3′) [26] primers, where Seq A and Seq B represent nucleotide sequences targeted by the second PCR primers. Then, randomized sequences of 0–5 bases were inserted into the mixed primers. PCR amplification was performed under the following conditions: denaturation at 94 °C for 30 s, annealing at 55° C for 30 s, and extension at 72° C for 30 s for 30–35 cycles. Fragments of the 16S rDNA PCR products were amplified again using additional PCR forward (5′- Adaptor C–Tag sequence—Seq A -3′) and reverse primers (5′- Adaptor D—Seq B -3′), where Adaptors C and D were used for the MiSeq sequencing reaction. The Tag sequence included 8 nucleotides designed for sample identification barcoding. Thermal cycling was performed under the following conditions: denaturation at 94 °C for 30 s, annealing at 60 °C for 30 s, and extension at 72 °C for 30 s for 10 cycles. PCR amplicons from each sample were used for high-throughput sequencing on a MiSeq Genome Sequencer (Illumina, San Diego, CA, USA). The obtained reads that had a quality value score of ≥ 20 and a length of >150 bases were extracted using the FASTX-Toolkit [27]. The paired-end reads were concatenated using a paired-end merge script, FLASH [28].

Next, chimeric sequences were deleted using the UCHIME algorithm of USEARCH [29]. In QIIME, the chimeric-filtered sequences were clustered into operational taxonomic units (OTUs) based on having >97% similarity with sequences in the Greengenes database [30]. The OTUs were tabulated on each taxonomic level from phylum to genus and their relative abundances were calculated using a workflow script in QIIME. The microbial α-diversity and β-diversity were calculated from the unweighted and weighted UniFrac distance matrixes using a script in QIIME. All of the above procedures were performed by Bioengineering Lab. Co., Ltd. (Kanagawa, Japan).

### Statistical analysis

Data are expressed as mean ± standard error (SE). Differences among experimental dietary groups were analyzed by Tukey–Kramer’s or Steel–Dwass’s multiple comparison test. All statistical analyses were performed using JMP Pro (Version 13.0, SAS Institute Inc., Cary, NC, USA). In all analyses, a two-sided p-value <0.05 was considered significant.

## Results

### Food intake, body weight, and organ weights

**Fig 1:**
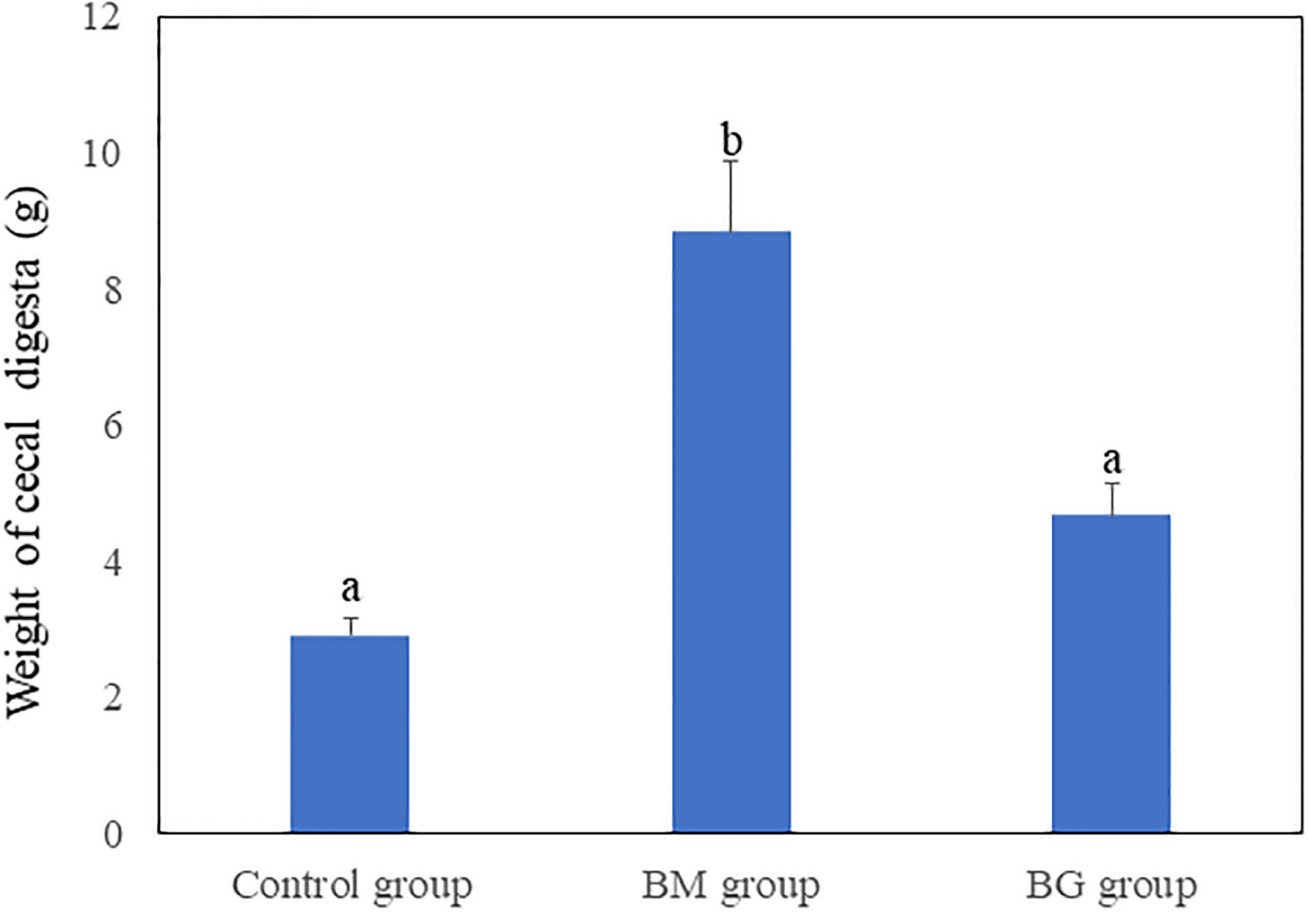
Comparison of weight of cecal digesta in rats fed the test diets. Bars represent the mean and standard error (SE). Means with different superscript letters differ significantly (Steel–Dwass test, p < 0.05). Control group, group fed the control diet; BM group, group fed the BARLEYmax diet; BG group, group fed the high-β-glucan barley diet.

There were no significant differences in final body weight, body weight gain, or food efficiency ratio among the three groups (Table 3). Food intake in the BM group was significantly lower than that in the control group, whereas there were no significant differences in food intake between the BG group and either of the other two groups. The weights of the cecal digesta of the rats fed the test diets are shown in Fig 1. The weight of the cecal digesta was significantly higher in the BM group than in the BG and control groups. The weight of the liver was not significantly different among the groups (data not shown).

**Table 3:** Final body weight, body weight gain, food intake, and food efficiency ratio of rats fed the test diets.

	Control group	BM group	BG group
Initial body weight (g)	174 ± 8.5	173.7 ± 7.0	174.2 ± 8.3
Final body weight (g)	415.9 ± 27.9	388.2 ± 50.0	402.1 ± 36.3
Body weight gain (g/day)	8.6 ± 0.8	7.7 ± 1.6	8.1 ± 1.1
Food intake (g/day)	22.7 ± 1.6^a^	19.5 ± 2.4^b^	20.8 ± 1.6^a^^b^
Food efficiency ratio (%)*	38 ± 1.2	28.9 ± 3.7	38.9 ± 32

Values are expressed as means ± standard error (SE) (n = 8).

*Food efficiency ratio = Body weight gain / Food intake × 100

In a row, means with a different superscript letter are significantly different (Tukey–Kramer’s test, p < 0.05).

### SCFA concentrations in the cecal, proximal colonic, and distal colonic digesta

The concentrations of SCFAs in the cecal digesta of the experimental rats are shown in Table 4. The concentrations of acetate, *n*-butyrate, and total SCFAs in the cecal digesta were significantly higher in the BM and BG groups than in the control group. The concentrations of SCFAs in the proximal and distal colonic digesta of the rats are shown in Table 5. There were no significant differences in the SCFA concentrations in the proximal colonic digesta among the three groups. The concentrations of acetate and total SCFAs in the distal colonic digesta of the rats were significantly higher only in the BM group than in the control group, whereas significant differences were not observed between the BG group and either of the two other groups. The proximal colonic digesta had the lowest quantities of SCFAs among the cecal, proximal colonic, and distal colonic digesta.

**Table 4:** Short-chain fatty acid (SCFA) concentrations in the cecal digesta of rats fed the test diets.

SCFA (μmol/g cecum)	Cecal digesta		
	Control group	BM group	BG group
Acetate	6.42±1.18^a^	18.25±3.32^b^	13.28±2.18^b^
Propionate	4.14±0.62	6.32±1.27	6.63±0.87
*n*-Butyrate	2.53±0.35^a^	11.15±2.78^b^	10.29±2.49^b^
Other SCFAs*	14.19±2.77	17.88±5.94	7.17±2.68
Total SCFA	27.29±4.33^a^	53.60±8.78^b^	37.37±4.28^a^^b^

All values are expressed as mean ± SE, n = 8 in all groups.

*Other SCFAs, the sum of the concentrations of formate, iso-butyrate, iso-varerate, and varerate is shown.

In a row, means with a different superscript letter are significantly different (Steel–Dwass’s test, p < 0.05).

Control group, group fed the control diet; BM group, group fed the BARLEYmax diet; BG group, group fed the high-β-glucan barley (BG012) diet.

**Table 5:** Short-chain fatty acid (SCFA) concentrations in proximal and distal colonic digesta of rats fed the test diets.

SCFA(μmol/g colon digesta)	Proximal colonic digesta			Distal colonic digesta		
	Control group	BM group	BG group	Control group	BM group	BG group
Acetate	0.50±0.11	0.95±0.30	0.91±0.46	2.09±0.22^a^	6.32±0.73^b^	6.49±2.16^a^^b^
Propionate	0.88±0.20	0.41±0.11	0.80±0.32	2.08±0.27	3.77±0.93	6.07±2.02
*n*-Butyrate	nd.	0.05±0.03	nd.	2.20±0.37	2.70±0.44	2.69±1.81
Other SCFAs*	0.05±0.03	nd.	nd.	3.64±0.64	13.80±4.57	4.61±2.53
Total SCFA	1.42±0.33	1.47±0.38	1.72±0.62	10.03±0.91^a^	26.59±4.93^b^	19.85±6.21^a^^b^

All values are expressed as mean ± SE, n = 8 in all groups. nd.: not detected.

*Other SCFAs, the sum of the concentrations of formate, iso-butyrate, iso-varerate, and varerate is shown.

In a row, means with a different superscript letter are significantly different (Steel–Dwass’s test, p < 0.05).

Control group, group fed the control diet; BM group, group fed the BARLEYmax diet; BG group, group fed the high-β-glucan barley (BG012) diet.

### Cecal and distal colonic microbiomes

The relative abundances of bacterial phyla in the cecal and distal colonic digesta of rats are shown in Table 6. The abundance of Bacteroidetes in cecal digesta was significantly higher in the BM group than in the control group, and tended to be higher in the BG group than in the control group (*p* = 0.06).

**Table 6:** Relative abundances of bacteria phyla in cecal and distal colonic digesta.

Phylum	Cecal digesta			Distal colonic digesta		
	Control group	BM group	BG group	Control group	BM group	BG group
Actinobacteria	0.85±0.27	3.64±0.55	2.94±1.26	0.21±0.05	2.98±0.67	2.35±1.20
Bacteroidetes	6.48±4.10^a^	20.80±2.94^b^	18.14±4.30^a^^b^	16.96±2.39^a^	24.72±1.37^a^^b^	26.26±2.97^b^
Firmicutes	83.20±5.33^a^	50.90±3.65^b^	63.28±7.80^b^	76.87±3.21^a^	52.68±2.93^b^	57.62±4.11^b^
Proteobacteria	1.21±0.45^a^	20.37±1.86^c^	7.77±1.58^b^	1.70±0.41^a^	12.45±1.31^b^	10.48±2.18^b^
Tenericutes	2.99±0.92^a^	0.00±0.00^b^	0.25±0.14^b^	0.94±0.37^a^	0.02±0.01^b^	0.04±0.02^b^
Verrucomicrobia	4.82±1.27	4.27±1.10	7.58±2.33	2.98±0.73	7.13±2.52	3.22±1.01

The percentages of the indicated bacteria phylum among all detected bacteria phyla are shown.

All values are expressed as mean ± SE, n = 8 in all groups.

In a row, means with a different superscript letter are significantly different (Steel–Dwass’s test, p < 0.05).

Control group, group fed the control diet; BM group, group fed the BARLEYmax diet; BG group, group fed the high-β-glucan barley (BG012) diet.

In contrast, the abundance of Firmicutes in cecal digesta was significantly lower in the BM and BG groups than in the control group (Table 6). In distal colonic digesta, the abundance of Bacteroidetes was significantly higher in the BG group than in the control group, and tended to be higher in the BM group than in the control group (*p* = 0.07). The abundance of Firmicutes in distal colonic digesta was significantly lower in the BM and BG groups than in the control group. The abundance of Proteobacteria in distal colonic digesta was significantly higher in the BM and BG groups than in the control group. The abundance of Proteobacteria in cecal digesta was significantly higher in the BM group than in the BG group.

The relative abundances of selected bacterial genera in the cecal and distal colonic digesta of rats are shown in Table 7. The abundances of *Bifidobacterium* and *Sutterella* in the cecal and distal colonic digesta were significantly higher in the BM and BG groups than in the control group. The abundance of *Ruminococcus* in cecal digesta was significantly lower in the BG group than in the control group, and that in distal colonic digesta was significantly lower in the BM and BG groups than in the control group.

**Table 7:** Relative abundances of selected bacteria genera in cecal and distal colonic digesta from rats fed the test diets.

Genus	Cecal digesta			Distal colonic digesta		
	Control group	BM group	BG group	Control group	BM group	BG group
*Bifidobacterium*	0.01 ± 0.00^a^	3.21 ± 0.54^b^	2.27 ± 1.25^b^	0.00 ± 0.00^a^	2.66 ± 1.85^b^	1.92 ± 1.16^b^
*Lactobacillus*	7.48 ± 1.82	11.47 ± 6.67	12.88 ± 1.53	16.96 ± 2.39	24.71 ± 1.38	26.26 ± 2.97
*Akkermansia*	4.82 ± 1.27	4.27 ± 1.10	7.58 ± 2.33	2.98 ± 0.73	7.13 ± 2.52	3.21 ± 1.01
*Clostridium*	1.60 ± 0.37^a^	0.05 ± 0.06^b^	1.74 ± 0.87^a^	0.14 ± 0.04^a^	0.01 ± 0.01^b^	0.07 ± 0.03^a^^b^
*Parabacteroides*	6.48 ± 4.10^a^	20.80 ± 2.94^b^	18.14 ± 4.30^a^^b^	16.96 ± 2.39	24.71 ± 3.89	26.26 ± 2.67
*Sutterella*	0.34 ± 0.17^a^	20.20 ± 1.85^c^	7.46 ± 1.57^b^	1.12 ± 0.36^a^	11.11 ± 1.45^b^	9.60 ± 2.21^b^
*Blautia*	3.02 ± 0.49	4.41 ± 0.76	2.74 ± 0.91	4.22 ± 0.70	5.24 ± 1.09	2.87 ± 0.74
*Oscillospira*	1.83 ± 0.52	2.07 ± 0.43	1.56 ± 0.43	1.43 ± 0.16^a^	2.82 ± 0.44^b^	1.68 ± 0.37^a^^b^
*Ruminococcus*	3.96 ± 0.54^a^	2.70 ± 0.65^a^^b^	1.57 ± 0.3^b^	6.34 ± 0.84^a^	1.49 ± 0.19^b^	1.43 ± 0.35^b^
*Eubacterium*	1.07 ± 0.33	0.58 ± 0.15	0.79 ± 0.29	1.42 ± 0.52	1.04 ± 0.31	1.03 ± 0.31

The percentages of the indicated bacterial genus among all detected bacterial genera are shown.

All values are expressed as mean ± SE, n = 8 in all groups.

In a row, means with a different superscript letter are significantly different (Steel–Dwass’s test, p < 0.05).

Control group, group fed the control diet; BM group, group fed the BARLEYmax diet; BG group, group fed the high-β-glucan barley (BG012) diet.

The abundance of *Parabacteroides* in cecal digesta was significantly higher in the BM group than in the control group. The abundance of *Oscillospira* in distal colonic digesta was also significantly higher in the BM group than in the control group. In contrast, the abundances of *Clostridium* in the cecal and distal colonic digesta were significantly lower in the BM group than in the control group. Significant differences in the abundances of *Clostridium* in the cecal and distal colonic digesta between the BG and control groups were not observed. There were no significant differences in the abundances of other bacteria genera among the groups.

Comparisons of the diversity of microbiotas in the cecal and distal colonic digesta among the experimental groups are shown in Table 8. In the cecum, the phylogenetic diversity, observed number of OTUs, and Shannon index in the BM group were significantly lower than those in the BG group, and all indices of diversity of microbiota in the distal colonic digesta in the BM group were significantly lower than those in the BG group.

**Table 8:** Comparison of gut microbiota diversity between cecal and distal colonic digesta from rats fed the test diets.

Diversity index	Cecal digesta			Distal colonic digesta		
	Control group	BM group	BG group	Control group	BM group	BG group
Phylogenetic diversity (PD_whole_tree)	30.2 ± 2.1^a^^b^	23.3 ± 2.4^b^	28.9 ± 1.4^a^	38.0 ± 3.6^a^^b^	29.1 ± 0.9^b^	46.3 ± 1.2^a^
Chao1	1406 ± 119	988 ± 74	1202 ± 140	2407 ± 300^a^^b^	1597 ± 296^b^	2964 ± 380^a^
Observed number of OTUs (observed_species)	562 ± 53^a^^b^	389 ± 26^b^	574 ± 39^a^	808 ± 90^a^^b^	562 ± 29^b^	1037 ± 36^a^
Shannon index	4.8 ± 0.2^a^	3.8 ± 0.1^b^	5.3 ± 0.1^a^	4.7 ± 0.3^a^	4.2 ± 0.1^b^	5.3 ± 0.1^a^

All values are expressed as mean ± SE, n = 8 in all groups.

In a row, means with a different superscript letter are significantly different (Steel–Dwass’s test, p < 0.05).

Control group, group fed the control diet; BM group, group fed the BARLEYmax diet; BG group, group fed the high-β-glucan barley (BG012) diet.

## Discussion

We investigated whether supplementation with BM, which contains several types of fermentable dietary fibers including fructan, β-glucan, and resistant starch, modifies the distal colonic microbiota more favorably than supplementation with BG, which contains a high amount of β-glucan but lower amounts of fructan and resistant starch than BM.

A previous report showed the effects of the consumption of a diet containing *Himalaya 292* (which contains 5% of neutral non-starch polysaccharides), which was the previous cultivar name of BM, on large bowel SCFAs in rats as compared with several other cereal products [31]. These data indicated that *Himalaya 292* resulted in alterations in the concentrations of colonic SCFAs (mainly acetate) in rats compared with two hull-less standard barleys. This result was consistent with our results concerning the increase in the acetate concentration in distal colonic digesta. However, the β-glucan contents in the standard barleys used in the previous report [31] were not determined. Therefore, it is difficult to discuss the significance of fructan and resistant starch in BM. It has also been reported that *Himalaya 292* [in which the fiber content as non-starch polysaccharide (NSP) was set at 7.5%] altered indices of large bowel fermentation in pigs [32]. These data suggested that the influences of BM on distal colonic and large bowel anaerobic, aerobic, coliform, and lactic acid bacteria were relatively small, indicating a lack of a specific prebiotic action. Another report indicated that the consumption of *Himalaya 292*-supplemented foods (total dietary fiber intake 44.7g/day) resulted in higher excretion of butyrate, higher distal colonic total SCFA excretion, and a lower fecal *p*-cresol concentration as compared with several refined cereal foods in humans [33]. These results were consistent with our results concerning the increase in the total SCFA concentrations in distal colonic digesta. However, previous reports did not compare BM and another high-β-glucan barley line. The effects of β-glucan or fructan and resistant starch on colonic SCFA production were unknown. Our data supported that several fermentable fibers in BM reached the distal colon and increased the concentration of total SCFAs in the digesta of the distal colon more so than those in β-glucan-rich BG.

It has been reported that combining wheat bran with resistant starch had more benefits than did wheat bran alone [34]. This finding may have important implications for the dietary modulation of luminal digesta, especially in the distal colon. Our data showed that the proximal colonic digesta had the lowest quantities of SCFAs among the digesta of the sites examined (i.e., the cecum, proximal colon, and distal colon). It was suggested that SCFAs might be absorbed at a faster rate in the proximal colon than in the distal colon and cecum [35,36]. In the proximal colon, no differences in SCFA concentrations were observed among the three experimental groups. In contrast, SCFA concentrations were higher in the distal colonic digesta of rats fed the BM diet as compared to the control diet. BM contains various fermentable fibers such as fructan, β-glucan, and resistant starch, which have different fermentation rates. Fructans are rapidly fermentable, whereas resistant starches are fermented slowly. The monophasic model described by Groot *et al*. [37] indicated several fermentation parameters *in vitro* with a pig fecal inoculum by using the cumulative gas production technique to examine the kinetics of fermentation after 48 h. The relative fermentation speed was expressed as the time at which half of the asymptotic value has been reached (h). The relative fermentation speeds of fructan, β-glucan, and resistant starch were 8.4, 17.7, and 35.3 h, respectively [38,39]. Therefore, fructans and β-glucans may be fermented mainly in the cecum, whereas resistant starches may be fermented in the distal colon.

The cecal and distal colonic abundances of Bacteroidetes were higher in the BM and BG groups than in the control group. By contrast, the abundance of Firmicutes was lower in the BM and BG groups than in the control group. Bacteroidetes and Firmicutes are the major bacterial phyla in the colonic microbiota. Our results indicated that the β-glucan in BM and BG increased the abundance of Bacteroidetes and decreased the abundance of Firmicutes. It has been reported that diets with high-fiber complex carbohydrates enrich the abundance of Bacteroidetes and reduce the abundance of Firmicutes in human adults [40]. These results may indicate that the increase in the abundance of Bacteroidetes is a microbial marker of complex carbohydrates such as BM and BG. It has been reported that the induction of secretory IgA, which helps to neutralize the toxins produced by microbes and prevents adherence of the microbiota to the intestinal lumen, appeared to be more efficient in the presence of Bacteroidetes [41]. Therefore, the effects of BM and BG in increasing the abundance of the phylum Bacteroidetes can be expected to be beneficial for preventing colonic disorders.

The abundances of *Bifidobacterium* in the cecal and distal colonic digesta were significantly higher in the BM and BG groups than in the control group. It has been reported that dietary supplementation with fibers particularly involving fructans and galacto-oligosaccharides, resulted in higher abundances of *Bifidobacterium* spp. and *Lactobacillus* spp. as well as an increased fecal butyrate concentration as compared with placebo/low-fiber-supplemented diet groups in healthy adults [8]. Our results supported that the β-glucan in BM and BG increased the abundance of *Bifidobacterium*. The increase in the cecal content of acetate that we observed could be partly explained by the increase in the abundance of *Bifidobacterium* in the BM and BG groups.

Concerning cecal and distal colonic microbial diversity, almost all diversity indices in the BM group were significantly lower than those in the BG group. It has been reported that consumption of 5 g/d low-molecular-weight β-glucan only impacted the β-diversity but the consumption of 3 g/d low-molecular-weight β-glucan failed to alter the β-diversity in a human study [42]. Diversity indices might be influenced by the amount of β-glucan in the diet. Further research is needed to explain the differences in diversity indices between the BM and BG groups.

The abundance of *Parabacteroides* in cecal digesta was significantly higher in the BM group than in the control group, whereas significant differences in the cecal abundance of *Parabacteroides* between the BG group and either of the other two groups were not observed. A previous report showed that a shift in the composition of the intestinal microbial community toward beneficial bacterial genera such as *Parabacteroides* was effective for reducing intestinal epithelial inflammation [43]. *Parabacteroides* spp. influence T-cell differentiation by enhancing and maintaining IL-10-producing Treg cells [44]. Therefore, the effect of BM in increasing the abundances of *Parabacteroides* can be expected to be beneficial for preventing colonic inflammation and enhancing intestinal immunological functions.

The abundance of *Oscillospira* in distal colonic digesta was significantly higher in the BM group than in the control group, whereas significant differences in the cecal abundance of *Oscillospira* between the BG group and either of the other two groups were not observed. *Oscillospira* is an under-studied anaerobic bacterial genus from *Clostridial cluster IV* that has resisted cultivation for over a decade since it was first observed [45]. Very little is known about its metabolism and physiology. However, recent reports inferred that *Oscillospira* spp. are butyrate producers [44]. In the present study, the observed increase in the concentration of *n*-butyrate in distal colonic digesta might have been partly caused by the increase in the abundance of *Oscillospira*.

The abundances of *Sutterella* in the cecal and distal colonic digesta were significantly higher in the BM and BG groups than in the control group. The abundance of *Sutterella* in the cecal content was also significantly higher in the BM group than in the BG group. *Sutterella* is a genus belonging to the phylum Proteobacteria and has the characteristics of gram-negative, non-spore-forming rods [46]. Significant increases in the cecal and distal colonic abundances of Proteobacteria might be due to the increase in the abundances of *Sutterella*. *Sutterella* spp. are notably resistant to human bile acids, which may account for their survival in the biliary tract and bowel. The butyrate-producing ability of *Sutterella* is less clear [47]. Furthermore, *Sutterella* spp. promote a protective immunoregulatory profile *in vitro* [48]. The effects of BM in increasing the abundances of *Sutterella* may be expected to be beneficial for the host’s intestinal conditions, and this possibility needs to be investigated by further studies.

Our results indicated that both BM and BG increased the concentrations of acetate and n-butyrate in cecal digesta and the abundance of *Bifidobacterium* in cecal and distal colonic digesta. These changes were considered to be due to β-glucan fermentation. The BM diet increased the concentration of total SCFAs in cecal digesta and the concentrations of acetate and total SCFAs in the distal colonic digesta. These changes may have been caused by the fructan and resistant starch in addition to β-glucan in BM. In conclusion, fermentable dietary fibers in BM reached the distal colon and modified the microbiota from the cecum to the distal colon, leading to an increase in the concentration of total SCFAs in the distal colon contents, more effectively compared with the high-β-glucan barley line (BG).
